# Motion-robust myelin imaging in MRI using 1D projection gating

**DOI:** 10.1038/s41598-026-39238-4

**Published:** 2026-02-09

**Authors:** Jinil Park, Sam Sedaghat, Kader Karli Oguz, Youngkyoo Jung, Eddie Fu, Nian Wang, Fang Liu, Hyungseok Jang

**Affiliations:** 1 Department of Radiology, University of California, Davis, Sacramento, CA USA; 2 Department of Radiology, University Hospital Heidelberg, Heidelberg, Germany; 3 Advanced Imaging Research Center, University of Texas Southwestern Medical Center, Dallas, TX USA; 4 Athinoula A. Martinos Center for Biomedical Imaging, Massachusetts General Hospital, Charlestown, MA USA; 5 Harvard Medical School, Boston, MA USA

**Keywords:** Neuroscience, Physics

## Abstract

Myelin-weighted imaging with inversion-recovery ultrashort-echo-time (IR-UTE) is highly motion-sensitive, yet extra hardware or longer scans are impractical. We evaluated whether a 1D superior–inferior (SI) navigator with bit-reversed spoke-angles mitigates motion artifacts without extending acquisition. Dual-echo IR-UTE was implemented at 3T. After an adiabatic inversion pulse, 21 spokes were acquired per segment around the white-matter null point, and dual-echo subtraction suppressed residual long-T2 signals. Adding SI-navigators at the end of each segment allows motion detection without lengthening TR. And bit-reversal is used to pseudo-randomize the spoke-angles. Bloch simulations of a 2D synthetic brain removed 10% of spokes to mimic gating. Three volunteers were scanned: (i) sequential-ordering, no-motion; (ii) bit-reversed, no-motion; (iii) bit-reversed with deliberate head nods. The navigator rejected 1,280 of 12,000 spokes (10.7%) during nodding, and the same gating was reconstructed on motion-free data. Simulation showed coherent streaks for sequential ordering but an isotropic point-spread function for bit-reversal. In vivo, gating degraded only the sequential dataset. bit-reversal preserved subcortical and deep-white-matter detail. During intentional nodding, gating with bit-reversal enhanced myelin contrast, outperforming the image reconstructed from the ungated data. The SI-navigator plus bit-reversal enables effective motion gating without hardware or time penalty, supporting routine motion-robust myelin mapping.

## Introduction

Myelin is a dense layer of cell membrane that wraps around axons, produced by non-neuronal cells known as oligodendrocytes^1,2^. Structurally, it consists of a multilayered membrane with alternating lipid and protein layers, forming an insulating sheath around nerve fibers in both the white and gray matter of the brain, as well as in the spinal cord. This insulation boosts the speed of action potential conduction by approximately 100 times and reduces the refractory period by more than 30 times^3^. Collectively, these enhancements amplify the brain’s capacity for information processing by over 3,000 times, making myelin essential for normal neurological functions such as speech, coordination, and cognition^3–5^. When axons lose their myelin, they also lose saltatory conduction, mechanical protection, and metabolic support, which can lead to structural and functional damage^6–8^. Myelin fundamentally transforms how electrical signals are initiated and propagated in the nervous system, and its disruption plays a key role in the pathology of numerous neurological disorders, including multiple sclerosis (MS)^9^, traumatic brain injury (TBI)^10,11^, epilepsy^12^, and Alzheimer’s disease (AD)^13–15^.

Direct assessment of myelin integrity in both white and gray matter may be important for diagnosing and evaluating the prognosis of myelin-related disorders. However, the hydrogens (^1^H, the source of signal in MRI) in myelin have extremely short T2 relaxation times (T2* < ~0.3 ms at 3T) and therefore cannot be directly imaged using conventional MRI sequences, which typically have echo times (TE) of several milliseconds or longer^16–23^. As a result, conventional sequences provide only an indirect or qualitative assessment of myelin. Another challenge in directly imaging myelin is selectivity, where long T2 water is present in much higher concentrations in the brain and produces a signal that is approximately 10–20 times stronger than that of myelin^24^. Consequently, myelin is invisible on conventional MRI. Alternatively, indirect imaging techniques have been explored, including myelin water fraction (MWF) mapping^25,26^, magnetization transfer (MT) imaging^27^, and quantitative susceptibility mapping (QSM)^28^.

Recently, inversion recovery ultrashort echo time (IR-UTE) sequences have been investigated to directly detection of ultrashort-T2* components predominantly associated with myelin, while suppressing long-T2 water^29–32^. The IR-UTE technique has been validated in both TBI and MS, showing promise in detecting changes in myelin content. For example, Ma et al. demonstrated that IR-UTE enables quantitative myelin-weighted imaging in the mouse brain using a preclinical 3T MRI scanner, allowing detection of white matter demyelination following open-field low-intensity blast injury (a model of mild TBI)^33^. Jang et al. reported that the myelin signal detected by IR-UTE significantly correlates with disability in MS patients, as measured by the Expanded Disability Status Scale (EDSS)^34^. These findings suggest that IR-UTE may serve as a potential imaging-based biomarker for diseases involving myelin alterations.

Despite promising results, a key limitation of the current IR-UTE technique is its long scan time (~ 10 min). This extended duration is primarily due to the inversion recovery imaging scheme, which requires a long repetition time (TR) of approximately 1 s. Additionally, since myelin-weighted imaging typically yields low signal intensity, a longer scan time is often necessary to improve the signal-to-noise ratio (SNR). However, prolonged imaging increases the risk of motion-related artifacts, such as blurriness, ghosting, and streaking, caused by intra-scan head movement. These artifacts can significantly degrade the already low-SNR myelin-weighted images. The issue is further exacerbated in patients with neurological disorders, who often have impaired motor control and may struggle to remain still during lengthy scans.

Motion artifacts in MRI are addressed with either prospective or retrospective gating. Prospective gating acquires data only at predefined (or predicted) time points and relies on external devices, such as an electrocardiogram (ECG), respiratory bellows, or a motion-tracking camera, to continuously monitor physiological signals^35–37^. By contrast, retrospective gating acquires data continuously and then sorts/bins it by a physiological surrogate, making scan time more predictable and allowing the gating window to be adjusted flexibly after assessing data quality.

Physiological surrogates for retrospective gating may come from external sensors (ECG, bellows, motion cameras) or be derived from the MR data itself. Common options include direct current (DC) gating, 1D projection navigators, and focused navigation (fNAV)^38–41^. DC gating uses the k-space center signal (reflecting sum of signal intensity in the image) and thus lacks explicit spatial encoding, which limits its ability to track head motion. In contrast, a 1D superior–inferior (SI) projection provides a spatial profile along the SI axis and is widely used in 3D UTE lung MRI studies for respiratory gating.

In this study, we propose a motion-gated IR-UTE technique using 3D radial encoding incorporating bit-reversed view ordering to correct for motion-related artifacts and enhance myelin-weighted imaging, without requiring additional scan time. In the proposed approach, we estimate motion using a 1D SI projection navigator appended after each readout and pair it with bit-reversed view ordering to help maintain near-uniform k-space coverage under gating^32,42^.

## Methods

### IR-UTE with dual-echo subtraction

Myelin has a short T2* relaxation time and low proton density (i.e., density of ^1^H)^23,24^, resulting in an extremely low detectable MR signal. As a result, it is sensitive to noise, aliasing or streak artifacts, ghosting, and image reconstruction errors, any of which can degrade myelin delineation. In addition, the large dynamic-range gap between myelin and neighboring structures increases the risk of quantization errors that compromise signal.

**Fig. 1:**
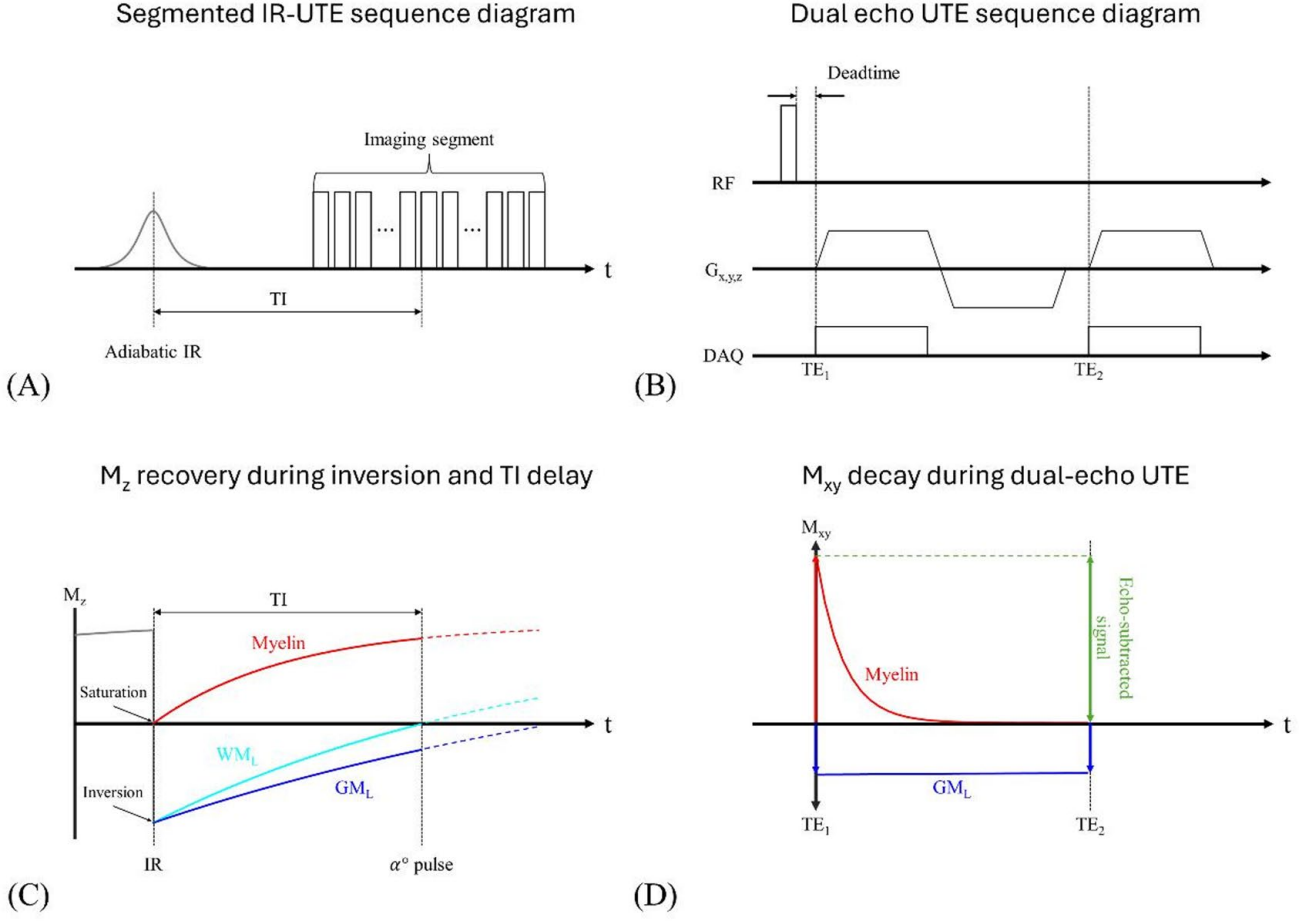
Sequence diagram and concept of the dual-echo IR-UTE technique used for myelin-weighted imaging. (**A**) Segmented IR-UTE sequence diagram. Non-selective adiabatic inversion pulse is followed by a TI delay chosen to null long-T2 white matter water (WM_L_). Each TR then acquires 21 radial spokes. (**B**) Dual-echo UTE read-out module. Spoke uses a hard pulse excitation and collects two ultrashort echoes. Centre-out gradients capture the first ultrashort echo (TE_1_ < < T2*), then rewind and re-ramp to sample a second echo (TE_2_). (**C**) Longitudinal-magnetization (M_z_) recovery during inversion and TI delay. Myelin (red) is driven to saturation (M_z_ ≈ 0) by the long adiabatic pulse and recovers fastest because of its short T1. Long-T2 white matter water (WM_L_, cyan) crosses zero at TI (dashed line), whereas long-T2 grey matter (GM_L_, blue) remains negative. (**D**) Transverse-magnetization (M_xy_) behavior during the dual-echo read-out at the TI tuned to null point of WM_L_. Myelin signal (red) decays rapidly between TE_1_ and TE_2_, while GM_L_ signal (blue) changes negligibly. Subtracting the two echoes therefore cancels long-T2 components and yields a myelin-specific image (green arrow).

To overcome these limitations, a method that combines an IR preparation pulse with UTE dual-echo subtraction has recently been proposed^31^. In this approach, an adiabatic inversion pulse is applied, followed by acquisition of two images at different TEs during the inversion time (TI) (Fig. 1A, B). Data are acquired with 3D center-out radial encoding using a monopolar fly-back dual-echo readout (Fig. 1B). Gradient-delay correction was performed through simple manual calibration of data acquisition windows, performed during sequence installation. The TI is selected so that the long-T2 white matter (WM_L_) is nulled (Fig. 1C). Because myelin’s T2* is extremely short, a long adiabatic inversion pulse cannot invert the magnetization; instead, it drives it to saturation (M_z_ ≈ 0)^43^. Immediately thereafter, the saturated myelin recovers rapidly owing to its short T1. At the chosen TI, the first echo captures signals of myelin, long-T2 grey matter (GM_L_), and any remaining long-T2 components, whereas the second echo is acquired after the myelin signal has substantially decayed (Fig. 1D). Subtracting the two echoes therefore isolates the myelin-weighted signal. Because the inversion recovery requires a long TR, acquiring one spoke per IR pulse significantly increases the scan time. To address this problem, the sequence acquires multiple k-space spokes segmented around the white matter null point, increasing acquisition efficiency (Fig. 1A).

### 1D SI projection navigating

This section describes our approach to motion tracking using 1D SI projection-navigating. Due to the oversampled center of k-space, non-Cartesian radial trajectories are known to be intrinsically more robust to motion than Cartesian trajectories. However, the motion can still affect the radial imaging, with unwanted image blurring, streaking, and ghosting. Therefore, identifying motion during scanning and discarding the motion-corrupted data can enhance the image quality. The need becomes even more critical when the subject makes an unexpected, bulk movement (e.g., patients with impaired motor function), after which the head may settle in a different position and appear severe artifacts. It is thus preferable to measure motion during the scan and address it retrospectively.

**Fig. 2:**
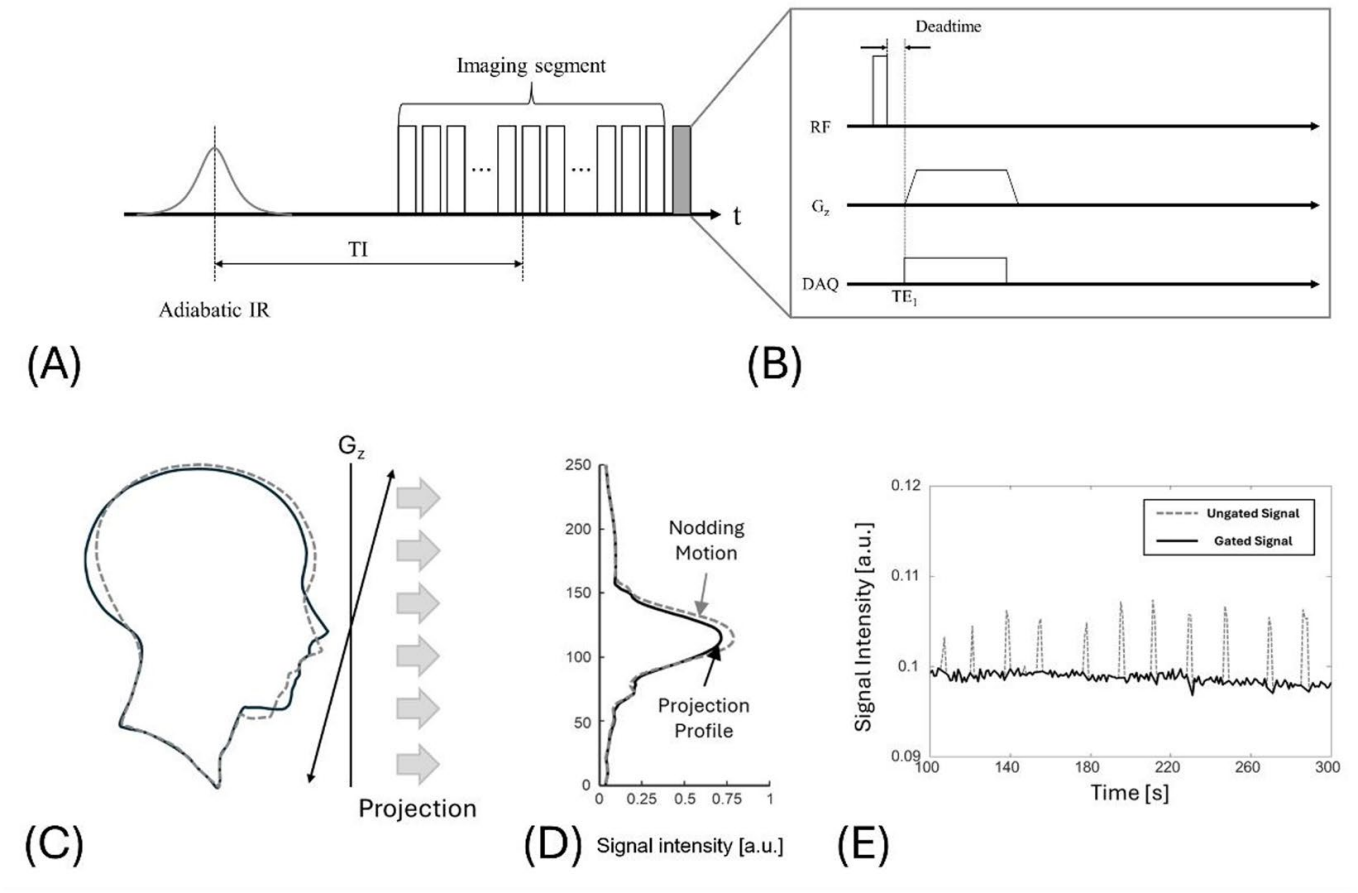

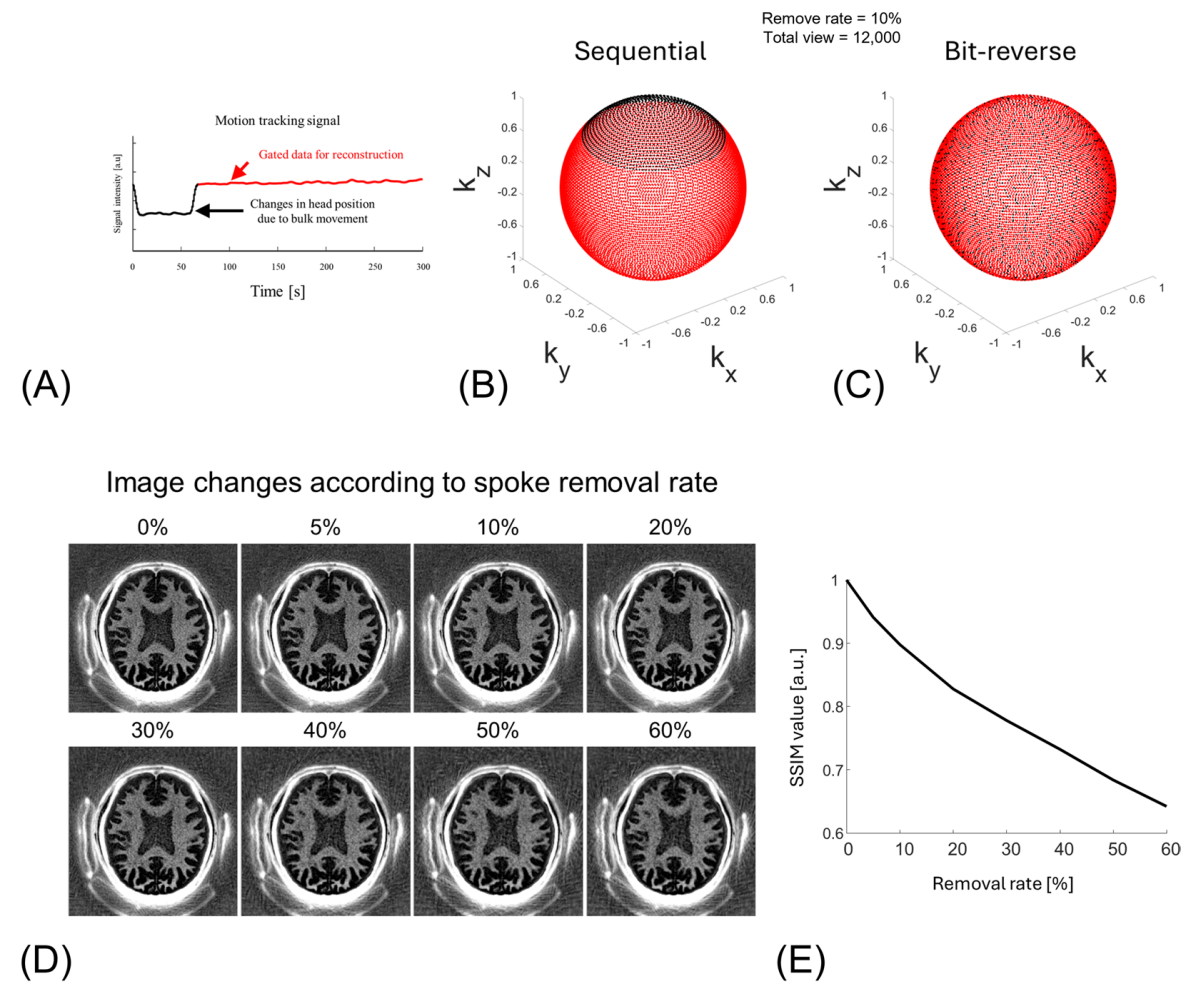
Superior–inferior (SI) navigator for motion gating in segmented IR-UTE. (**A**) Sequence diagram of the SI navigator used in the segmented IR-UTE sequence. Each IR segment consists of 21 image spokes and a single SI-navigator spoke (gray) at the end. (**B**) Enlarged view of the navigator spoke: a center-out read-out is applied along k_z_. (**C**) Head outline before (solid) and after (dashed) an intentional nod. (**D**) SI-projection profiles, showing profiles altered by intentional nods. (**E**) Navigator amplitude over time during the nodding experiment. The intentional head nods every 20 s are well represented (dashed line).

In the proposed approach, we monitor head motion using an SI projection navigator. The navigator is inserted at regular intervals to the end of each IR segment (Fig. 2A).

The navigator is placed at the end of segment maintains a consistent TI, ensuring stable contrast in motion tracking signal. This navigator, frequently used in 3D UTE lung MRI, periodically applies a short readout gradient along the G_z_ axis, acquires a half-projection k-space line (Fig. 2B), and then performs a 1D inverse Fourier transform to generate a SI projection profile (Fig. 2C and D)^40,44^. As a result, the projection profile captures comprehensive anatomical information of the head along the SI axis (Fig. 2C and D). Continuous comparison of the profiles enables tracking movement of head throughout the IR-UTE acquisition (Fig. 2D). Figure 2E shows an example of motion tracking using the 1D SI projection-navigator, estimated from the signal intensity at a fixed pixel location in the 1D profile, defined by the position of maximum signal at the first time point. In this example, the subject intentionally nodded their head every 20 s during the scan. When the head nods, the projection profile changes; this variation can be measured and incorporated into the image reconstruction process to reduce motion artifacts by excluding motion-contaminated data.

### Bit-reversed view ordering

In radial trajectories, it is important for retrospective gating to maintain that the remaining spokes are uniformly distributed in k-space after discarding unwanted data. If the spokes are acquired sequentially, the data loss caused by gating will be concentrated in a specific region of k-space, resulting in an undersampling artifact. Various sampling strategies such as golden-angle, phyllotaxis, and interleaved schemes have been proposed to avoid this problem^44–46^.

Here we adopt a bit-reversed view ordering. By reversing the binary index of each spoke, the original trajectory is rearranged into a pseudo-random sequence. For example, the 52nd spoke (binary 110100) becomes the 13th spoke (binary 001011) after the reordering. The main advantage is that it can be applied to any kind of trajectory, providing a pseudo-random view ordering.

### Simulation

A computer simulation was performed to test whether bit‑reversed view ordering can compensate for the k‑space non‑uniformity introduced by retrospective motion gating in IR‑UTE myelin-weighted imaging.

**Fig. 3:**
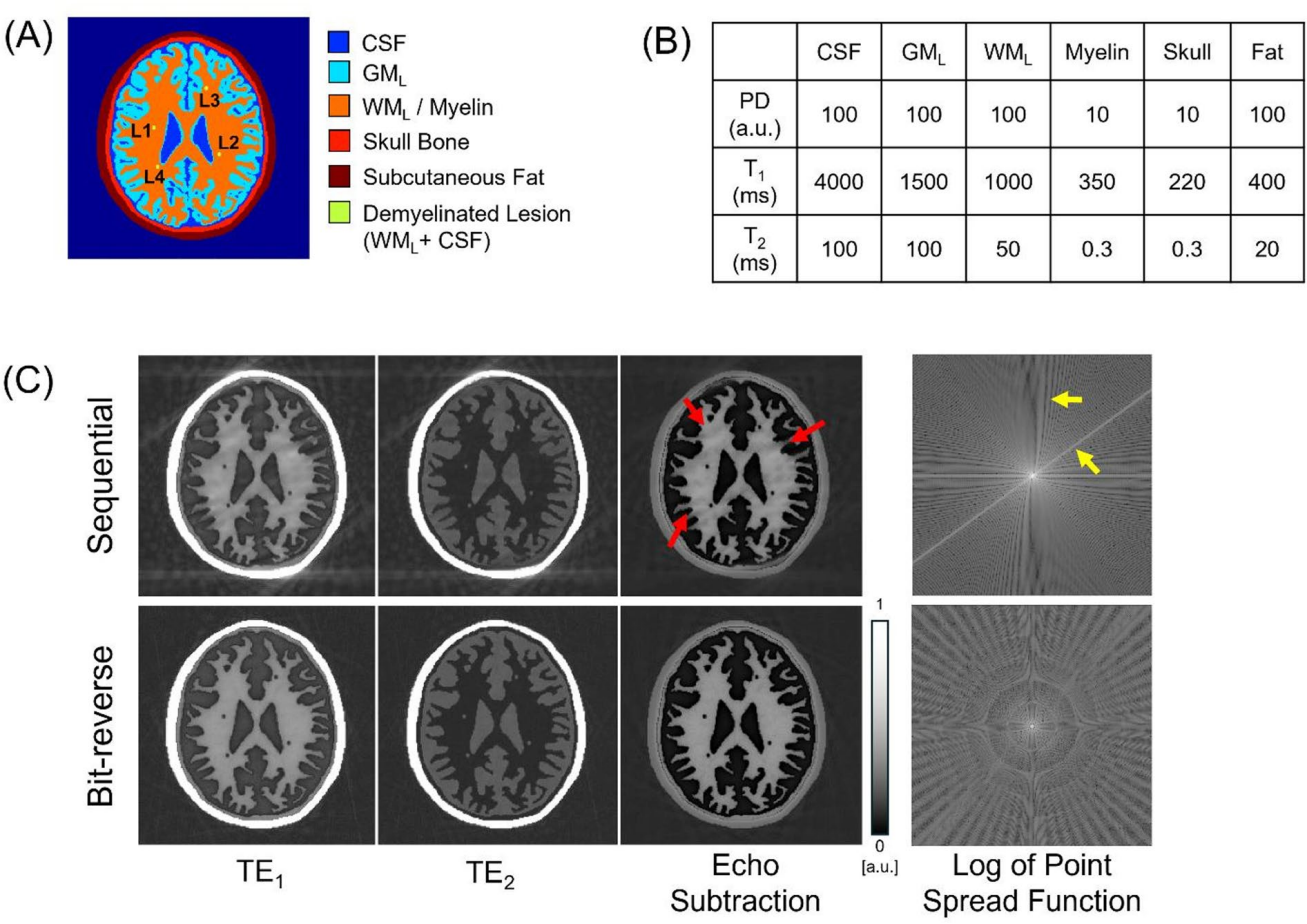
Simulation of motion-gating with sequential versus bit-reversed view ordering. (**A**) Two-dimensional digital brain phantom comprising cerebrospinal fluid (CSF), cortical grey matter (GM_L_), deep white matter with myelin (WM_L_/ Myelin), skull, subcutaneous fat, and a synthetic demyelinated lesion (L1–L4). (**B**) Proton-density (PD, a.u.), T1 (ms), and T2 (ms) values assigned to each tissue class. (**C**) Dual-echo IR-UTE images after deleting 69/692 [10%] of spokes to mimic retrospective gating. Sequential ordering produces coherent streaks and blurring in the myelin-weighted image (red arrows) and pronounced PSF side-lobes (yellow arrows), whereas bit-reversed view ordering disperses the missing spokes, yielding minimal artifacts and an isotropic PSF. Window/level were fixed across TE_1_, TE_2_, and echo-subtraction panels at 0-to-1. FOV [220 × 220 mm^2^], in-plane resolution [1 × 1 mm^2^].

A 2D high-resolution digital head phantom (654 × 654 pixels) consisting of six tissue components—cerebrospinal fluid (CSF), GM_L_, WM_L_, myelin within white matter, skull bone, and subcutaneous fat—was created, as illustrated in Fig. 3A. To construct the segmentation map, a sample brain map from the open-source segmentation tool, multiplicative intrinsic component optimization (MICO)^47^, was used, which provided labels for white and gray matters. The remaining three components (CSF, skull, and subcutaneous fat) were manually added. Four demyelinated lesions were placed within the white matter region. Using the segmentation map, a Bloch simulation was performed based on the tissue parameters shown in Fig. 3B. All simulations were conducted in MATLAB R2024a (MathWorks, Natick, MA) using in-house code that solves the Bloch equations analytically to model signal evolution.

The simulation reproduced an IR‑UTE dual‑echo acquisition, using the following sequence parameters: flip angle = 15°, TR = 1000 ms, inversion time TI = 379.9 ms (null point of WM_L_), readout bandwidth = 250 kHz, and field of view = 220 × 220 mm^2^, matrix size = 220 × 220. Two radial echoes were collected per spoke (TE_1_ = 0.03 ms, TE_2_ = 3 ms). A total of 692 spokes covered 2D circular k-space, delivered in segments of 21 spokes; the spoke‑to‑spoke interval was 6 ms. To assess sampling robustness, k‑space was traversed either sequentially or with bit‑reversed ordering. The data loss due to motion gating was assumed to be 10% of the total spokes.

### In vivo study

Three healthy male volunteers (42, 42, and 38-year-old) were scanned on a 3T clinical MRI system (MAGNETOM Prisma Fit, Siemens Healthineers, Erlangen, Germany) using a 32‑channel head/neck coil. A written informed consent was collected under an institutional‑review‑board (IRB) approval of University of California, Davis (Approval number: #2162542; Date: 2/28/2024). All procedures were performed in accordance with the Declaration of Helsinki and relevant institutional guidelines and regulations. Three scans were performed using 3D IR-UTE sequence: Sequential view ordering without motion (Scan #1), bit-reversed view ordering without motion (Scan #2), and bit-reversed view ordering with intentional motion (Scan #3). During Scan #3, the participant was instructed to nod their head once with a large movement. Scans #1 and #2 served as motion-free references, acquired using two different view ordering strategies, allowing for a controlled comparison of their effects under retrospective undersampling. To assess robustness to motion-induced data rejection, we identified and excluded motion-corrupted spokes in Scan #3 using a 1D SI-projection navigator with manual thresholding. The motion profile derived from Scan #3 was then reused to retrospectively remove 0–60% of spokes from Scans #1 and #2, simulating gating-induced undersampling with known ground truth.

All images were reconstructed in MATLAB R2024a using non-uniform fast Fourier transform (NuFFT) implementation (Kaiser-Bessel gridding kernel width = 3, β = 1.9, oversampling = 2.0)^48^. Density-compensation function (DCF) was estimated with an iterative scheme to improve k-space-weighting flatness^49^. Multi-channel data were combined using an iterative adaptive coil-combination method^50^. Via dual-echo processing, the myelin-weighted images were created by performing magnitude subtraction (TE_1_ - TE_2_) in image space. For quantitative analysis, we measured the contrast-to-noise (CNR) between myelin and GM_L_ on the myelin-weighted images. To assess image change due to motion and undersampling, we also computed the structural similarity index (SSIM) with respect to the fully sampled reference^51^. For Scan #3, we compared ungated and gated reconstructions by assessing sharpness using an edge rise distance (EDR) between 10% and 90%. TI was tuned near the WM_L_ null point under steady-state conditions (accounting for the effective shift with a finite TR and multiple spokes in a segment acquired per IR). In a fully recovered state, the WM_L_ null point lies around 550–693 ms, but under segmented steady-state acquisition (finite TR with multiple spokes per inversion) the effective WM_L_-null shifts. We therefore computed the steady-state WM_L_-null by Bloch simulation (accounting for TR, flip angle, and spokes per segment) and then empirically refined TI on the scanner to maximize WM_L_ nulling. The imaging parameters optimized through experimental fine-tuning and Bloch simulation are as follow: flip angle = 10°, TR = 1000 ms, TI = 320 ms (white matter null point tuned at 3T), dual echo readout (TE_1_ = 0.03 ms, TE_2_ = 2.2 ms), readout bandwidth = 92 kHz, field of view = 220 × 220 × 176 mm^3^, matrix size = 220 × 220 × 88, 20 spokes per segment, total spokes = 12,000, spoke to spoke interval = 5 ms, and scan time = 10 minutes. A 50 µs nonselective hard pulse excited each spoke, and a 10.24 ms hyperbolic secant inversion pulse preceded every segment. These choices reflect practical trade-offs. TR balances SNR and total scan time, and TI was adjusted through the simulation/in-scanner calibration process described earlier. A flip angle of 20° provides sufficient UTE SNR while limiting Mz drift within each segment, and 20 spokes per segment reduce scan time without excessive T1 blurring.

## Results

### Simulation

Bit-reversed view ordering significantly reduced artifacts caused by retrospective motion gating (Fig. 3C). When 10% of spokes were removed using sequential view ordering, prominent streaks appeared along the spoke directions, accompanied by blurring in myelin-rich regions (red arrows). In contrast, bit-reversed view ordering distributed the data loss more evenly, leading to a noticeable reduction in streaking artifacts. This improvement was also evident in the simulated point spread function (PSF): sequential view ordering resulted in pronounced side-lobes (yellow.

### In vivo study−1D SI projection navigator and gating

**Fig. 4:**
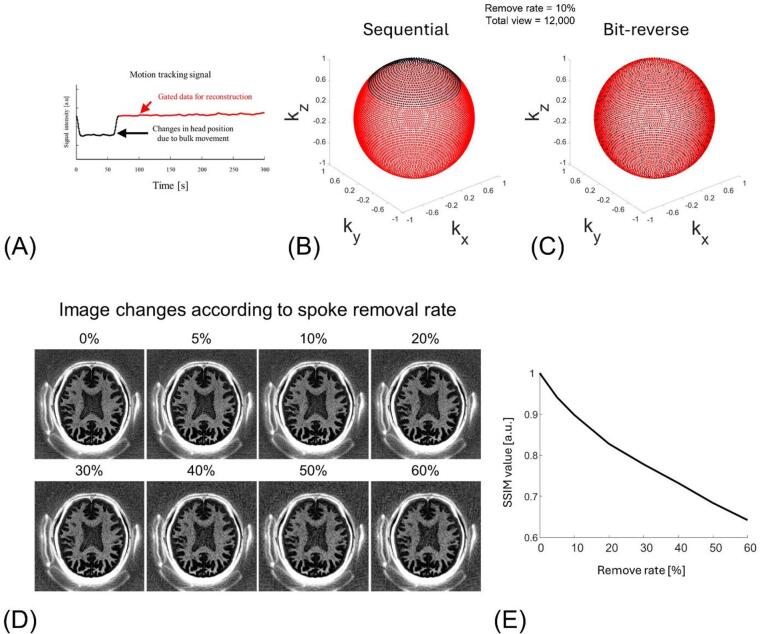
1D SI projection navigator gating signal (**A**) and the distribution of motion-gated spokes for sequential and bit-reversed view ordering, respectively (**B**, **C**). (**A**) Superior–inferior navigator signal obtained from the scan with intentional motion (Scan #3). Baseline shifts (black arrows) indicate large head nods. The motionless data are selected by the threshold gate and used for image reconstruction (black line, 10.7% of the 12,000 rejected spokes). (**B**) Endpoints of the gated(red) and rejected (black) spokes plotted on a unit sphere for sequential view ordering. Gating removes a contiguous sector, creating a large k-space part. (**C**) Corresponding distribution for bit-reversed view ordering. The same number of rejected spokes is pseudo-randomly dispersed, maintaining quasi-uniform k-space coverage. (**D**) Myelin-weighted IR-UTE images reconstructed after progressively removing 0%, 5%, 10%, 20%, 30%, 40%, 50%, 60% of spokes. Window/level fixed at -0.01-to-0.1 for all panels. FOV [240 × 240 mm2], in-plane resolution [1 × 1 mm2]. (**E**) structural similarity index (SSIM, a.u.) versus spoke-removal rate.

Figure 4A plots the navigator signal from Scan #3. The changed baseline reflects the changed head position due to intentional head nodding. Because Scan #3 involved a single instructed nod, the navigator exhibits two baseline plateaus separated by the motion event. We therefore used threshold-based gating to select the longer, more stable plateau (i.e., the interval containing more spokes) for reconstruction, which effectively suppressed motion artifacts. IR-UTE acquired 12,000 spokes and rejected 1,280 (10.67%) of them, which were head position data changed by nodding. The spokes where head position data changed by nodding are removed (black dots) are continuously missing in sequential view ordering (Fig. 4B), while bit-reversed view ordering scatters the missing segments pseudo randomly throughout the sphere (Fig. 4C). Figure 4D shows that image quality degrades as a larger fraction of motion-contaminated spokes is rejected, with increased blurring. Quantitatively, when approximately 60% of spokes were discarded, the SSIM remained around 0.65 (Fig. 4E).

### In vivo study–myelin-weighted imaging

For all three volunteers, the 1D SI projection-gating approach significantly improved the quality of myelin imaging in the presence of intentional head motion.

**Fig. 5:**
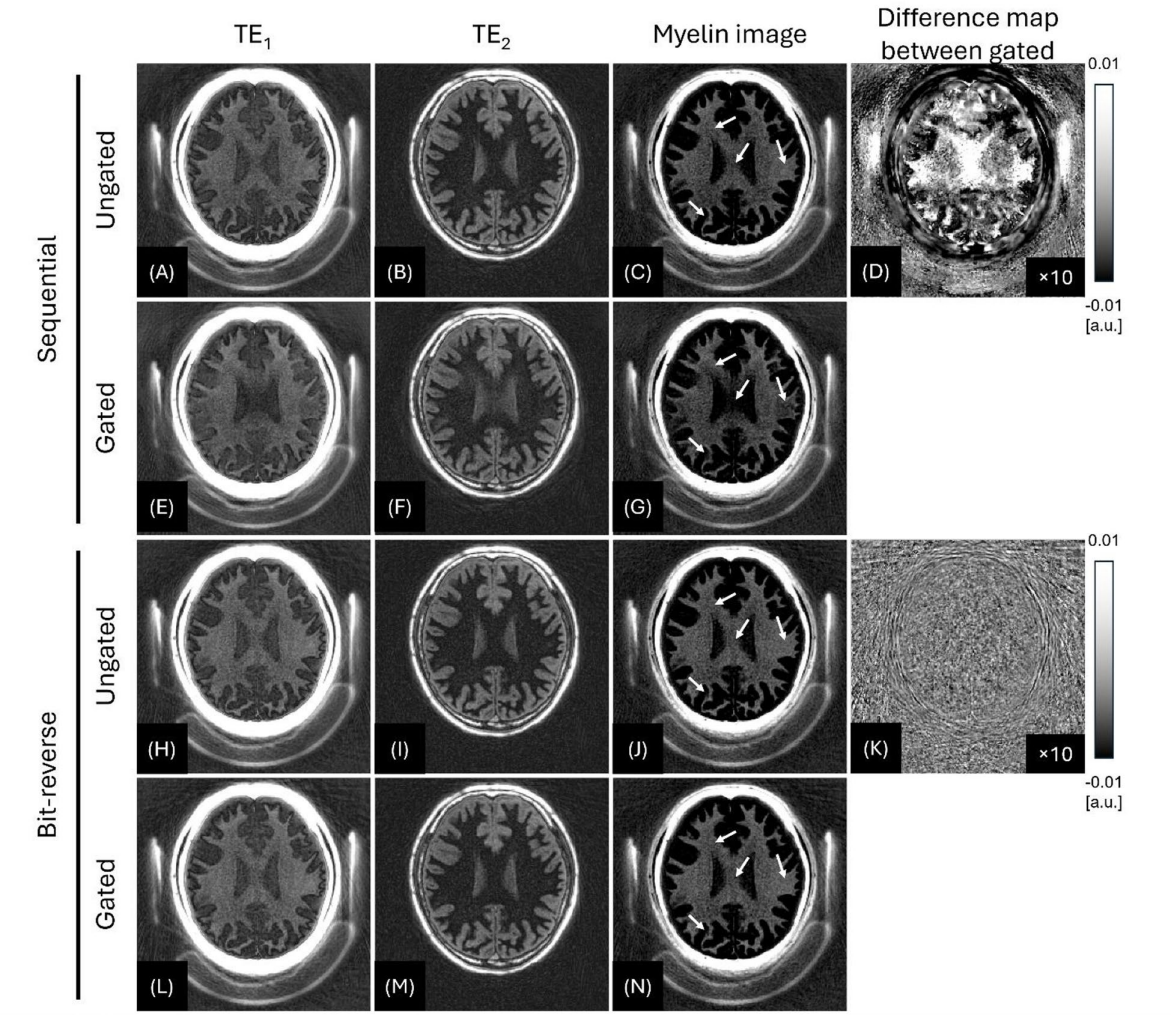
Effect of retrospective motion gating on sequential versus bit-reversed IR-UTE imaging (42-year-old male). Sequential view ordering case is shown in the upper two rows (Scan #1, **A-G**) and bit-reversed ordering case is shown in the lower two rows (Scan #2, **H-N**). Within each data, the first row shows the ungated (fully data) reconstruction, and the second row shows the gated (undersampled) reconstruction obtained after discarding 1,280 of 12,000 spokes (10.7%) identified by the navigator trace from Scan #3. Sequential view ordering produces focal signal alteration (**G**, white arrow), whereas the bit-reversed view ordering yields only low-level, incoherent noise (N, white arrows), which is also confirmed by difference images (between gated and ungated) shown in **D** and **K**. window/level were fixed across all panels at -0.01-to-0.1. FOV [240 × 240 × 176 mm^3^], in-plane resolution [1 × 1 × 2 mm^3^]. The difference maps (**D**,** K**) show (gated − ungated), displayed with a ×10 intensity scale-factor for visibility.

Figure 5 contrasts the myelin-weighted images from the two motion-free reference data (Scan #1 and Scan #2) obtained with sequential view ordering or bit-reversed view ordering, which were undersampled using a simulated motion-gating trace (obtained from Scan #3). This demonstrates how sequential and bit-reversed view orderings influence the myelin-weighted imaging, by comparing the images from ungated (fully sampled) and gated (undersampled) data. With sequential view ordering, the simulated gating produced focal signal loss in myelin (white arrows in Fig. 5C and G). In the corresponding difference image between ungated and gated myelin-weighted images (Fig. 5D), the error appears as coherent signal change across the brain. In contrast, bit-reversed view ordering improves the myelin-weighted image in both subcortical and deep-white-matter regions (white arrows in Fig. 5J and N). The corresponding difference image exhibits incoherent low-level noise, underscoring the scheme’s superior robustness (Fig. 5K).

Quantitatively, the SSIM between fully sampled and gated images was 0.960 for sequential ordering (Scan #1) and 0.992 for bit-reversed ordering (Scan #2), indicating better structural preservation with the latter under ~ 10% spoke rejection. CNR between myelin and GM_L_ also degraded less with bit-reversed ordering: for sequential, CNR decreased from 10.45 (fully sampled) to 9.65 (gated; ≈7.66% drop), whereas for bit-reversed it decreased from 10.75 to 10.67 (≈ 0.76% drop), indicating a smaller loss of contrast under gating.

**Fig. 6:**
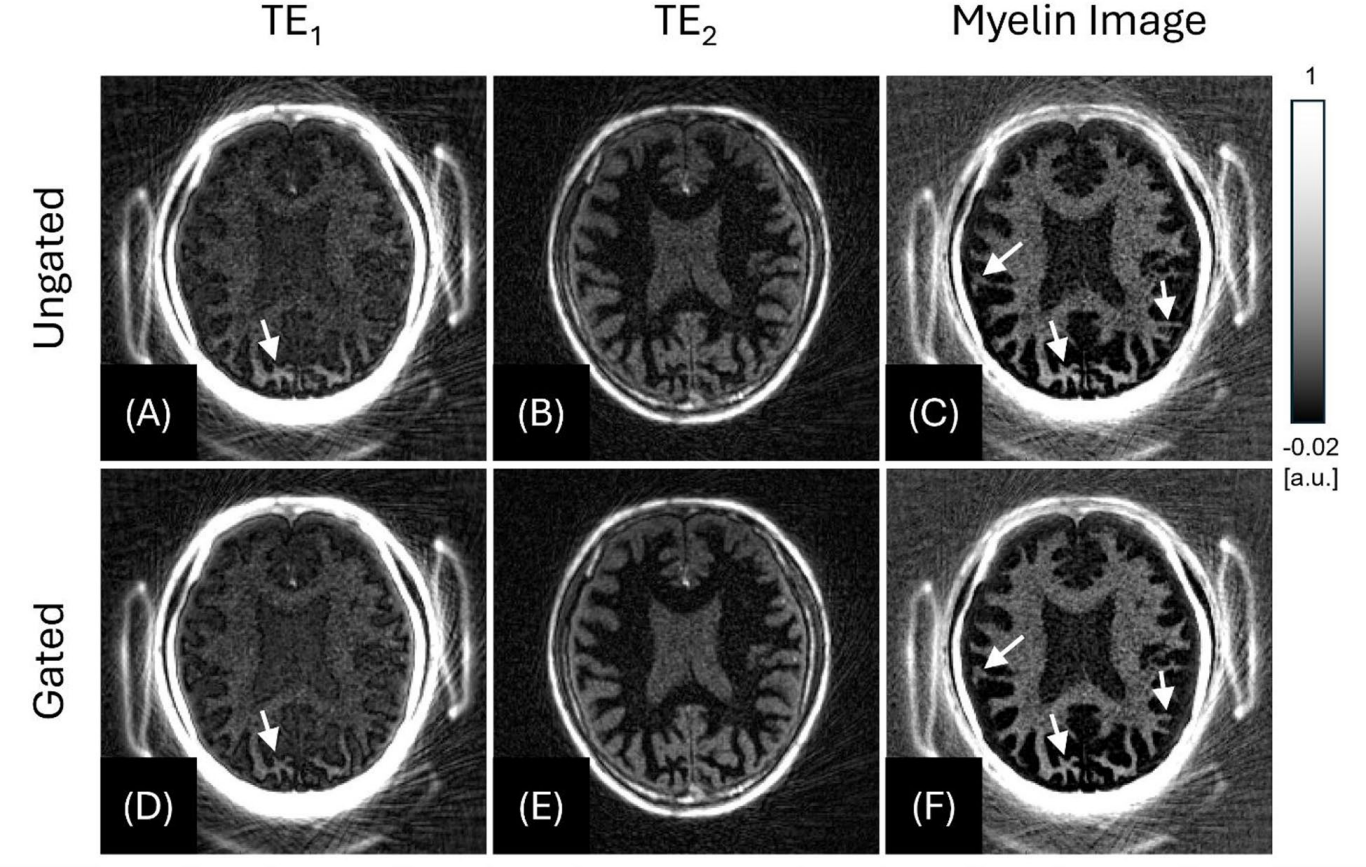
Effect of 1D SI projection-gating in the in vivo experiment with intentional head motion (Scan #3 with a 42-year-old male). Top row (**A–C**) shows the results from the full 12,000 spokes, without motion gating. Bottom row (**D–F**) shows the results after discarding the 1,280 of 12,000 spokes (10.67%) selected by the SI navigator. White arrows highlight artifacts caused by the head nods, which are conspicuous in the ungated images (**C**) but largely suppressed after gating (**F**). window/level were fixed across all panels at -0.02-to-0.1. FOV [240 × 240 × 176 mm^3^], in-plane resolution [1 × 1 × 2 mm^3^].

Figure 6 shows the myelin-weighted imaging with the intentional head motion (Scan #3), where 1D SI projection -gating and bit-reversed view ordering were utilized. Figure 6A and C were reconstructed from all acquired spokes without motion gating. Although the full data typically allows higher SNR than the undersampled data, the head nods may introduce blurring and ghost artifacts, impairing overall image quality and myelin contrast. Figure 6D and F show the result from the same data after retrospective motion gating. Spokes coinciding with nodding motion, detected by 1D SI projection -gating, were discarded before reconstruction. The CNR between myelin and GM_L_ showed a CNR of 6.91 for the ungated data and 8.32 for the gated data, and the EDR values for sharpness were 3.54 ± 0.57 for the Ungated data and 2.57 ± 0.49 for the gated data. Despite the reduced number of data sampling, sharper myelin boundaries and unbiased myelin signal were achieved by omitting the motion-contaminated data (white arrows).

## Discussion

Myelin has extremely short T2s (< 1 ms), due to its highly ordered microstructure and chemical shift with multiple lipid peaks. Therefore myelin cannot be directly imaged with conventional MRI sequences, which typically have TEs of several milliseconds or longer^16–23^. As a result, conventional sequences only provide an indirect assessment of myelin. The MR properties of myelin have been investigated by several groups. Broad line proton spectroscopy studies have demonstrated that myelin is in a liquid-crystalline state^16^. Multi-component analysis of spin echo or free induction decay of white matter samples has shown a broad range of short T2 or T2* values for myelin protons (e.g., ~ 50 µs^52^, 7.5–101 µs^53^, 50-1000 µs^18^, 150–250 µs^24^, etc.). UTE MRI sequences with TEs as short as ~ 8–32 µs, which are 100–1000 times shorter than those of conventional clinical sequences, allow direct detection of signal from myelin^54–56^.

Because the myelin signal is significantly lower than that of long T2 tissues in the brain, myelin-weighted imaging techniques rely on IR preparation and echo subtraction to suppress the long T2 signal. Despite the effectiveness of this suppression, the inherently low SNR of the myelin lipid remains a major challenge. In this context, even minor imaging artifacts can severely impact the detection of myelin. Unlike conventional Cartesian MRI, where motion typically results in ghosting artifacts along the phase-encoding direction, UTE imaging, based on a radial trajectory, produces motion artifacts that radiate in all directions. As a result, strong short T2 signals from surrounding bone or fat, as well as residual long T2 signals, can interfere with the detection of the myelin signal if motion occurs during the scan.

In this study, we demonstrated the efficacy of motion-gating using 1D SI projection-navigator and bit-reversed view ordering in IR-UTE for improved myelin-weighted imaging. The 1D SI projection-navigator does not impose any additional scan time because it is located at the end of segments (Fig. 2A), making it clinically feasible. Our findings show that combining SI 1D SI projection-navigator with a bit-reversed radial trajectory markedly improves the motion robustness of IR-UTE myelin-weighted imaging. In the motion-free volunteer, both ordering schemes scanned, when the navigator-derived gating trace was applied retrospectively the sequential data developed focal signal decrease, whereas the bit-reversed view ordering data retained cortical detail and myelin contrast (Fig. 5). When intentionally nodded, about 10% of the spokes were removed by the navigator. nevertheless, the gated, bit-reversed view ordering reconstruction recovered sharp enhanced grey/white contrast, outperforming the ungated image that had the full data set (Fig. 6). These results underscore two practical points: (i) dispersing missing spokes pseudo-randomly with bit-reversal converts coherent streaks into low-energy noise, and (ii) placing the SI navigator at each segment boundary permits effective gating without extending scan time. Because both modifications require only a gradient-table permutation and a single 1D readout, they can be deployed on existing clinical scanners and may enable reliable, motion-tolerant myelin mapping in routine neuroimaging.

Bit-reversing is a simple yet powerful method for achieving pseudo-randomized view ordering^42^. In IR-prepared imaging, this approach can improve image quality, particularly when a large number of spokes are acquired per inversion recovery, by redistributing T1 blurring effects in a pseudo-random manner. For example, multiple spokes may be acquired near the nulling point of long T2 white matter, with roughly half acquired before the null point and half after. Spokes acquired before the null point capture negative longitudinal magnetization (M_z_), while those acquired after the null point capture positive M_z_ from the long T2 white matter. Each spoke samples a different M_z_ magnitude, governed by the TI and T1 relaxation. Consequently, the encoded k-space can exhibit structured modulation patterns as a function of both T1 recovery and view ordering, which may degrade the PSF. Previous studies have explored variable flip angle excitation to compensate for this kind of signal variation^57,58^. However, determining optimal flip angles based on the long T2 white matter signal is not possible in the myelin-weighted imaging, as the target signal is ideally zero at the nulling point. As an alternative, bit-reversed view ordering randomizes these modulation patterns, making them more ‘noise-like’ and thereby reducing coherent artifacts. Further in-depth investigation of this approach will be pursued in future studies.

This study has several limitations. First, only three healthy volunteers were included to validate the proposed approach. Although the volunteers were instructed to make intentional motions during the scan, this may not accurately represent the conditions seen in patient groups with various neurological disorders. These constraints in sample size and study design also precluded quantitative repeatability analyses (e.g., based on Dice coefficient or IoU of segmented white matter structures and CNR). In future work, we will evaluate the sequence in healthy volunteers as well as patients with a range of neurological diseases and conduct repeatability tests under more natural, multi-directional motion. Second, we did not compare our method with alternative view ordering schemes, such as the golden angle method^59^. While the golden angle approach has demonstrated effectiveness in radial undersampled and time-resolved imaging, our goal is to distribute motion-contaminated spokes retrospectively. Future research will explore the impact of different ordering schemes. Third, we only incorporated an SI navigator, which was capable of detecting nodding motion. One 1D SI projection was acquired per TR, so the method was designed to detect changes in head position rather than to finely track subtle, continuous motion in real time. Future studies will investigate whether adding additional navigators along the Gx and Gy axes can more sensitively detect head motion in other directions, such as shaking and rotation, without increasing scan time, and to determine the reproducibility of the method. Fourth, other than simple delay correction, our IR-UTE imaging omitted B0 off-resonance compensation and advanced eddy current correction techniques^60,61^ that may further reduce blurring and streaking artifacts. The impact of these corrections will be investigated in future work. Additionally, because both echoes were acquired with monopolar readout gradients, which create nearly identical eddy currents, we did not perform echo-to-echo alignment. However, explicit image registration may be requisite for bipolar radial dual echo imaging or acquisitions with longer gradient echo trains^62^. Fifth, although inversion recovery and dual-echo subtraction substantially attenuate long-T2 water, absolute specificity to myelin may not be guaranteed because bound water/proteins, magnetization transfer (MT) effects, mineralization/iron, and tissue interfaces can contribute. As future work, we will validate IR-UTE myelin-weighted imaging in phantoms and specimens by comparing it with other techniques, such as quantitative MT^63^ and QSM^28^, to better understand the contribution of the non-myelin ultrashort-T2* sources in the brain.

## Conclusion

This study demonstrates the efficacy of motion gating using 1D SI projection-navigator and bit-reversed view ordering in IR-UTE for motion-robust myelin-weighted imaging. Bit-reversed view ordering is a one-line permutation of the gradient list that pseudo-randomizes spoke positions and keeps k-space nearly uniform even after data are rejected. The proposed approach demands no additional hardware, no extra scan time, but only minor modification on sequence, and hence it can be readily implemented on current clinical 3T systems. Combined with myelin-weighted imaging technique, the proposed approach could benefit patient groups prone to motion such as children, elderly, or epilepsy cohorts.

## Data Availability

The datasets used and/or analyzed during the current study available from the corresponding author on reasonable request.
